# Supplementary material for: Electroencephalography-Derived Functional Connectivity in Sensorimotor Networks in Post Stroke Fatigue
Source: Brain Topogr. 2023 Jun 17;36(5):727–35. doi: 10.1007/s10548-023-00975-8 (PMC10415526; doi:10.1007/s10548-023-00975-8)
Supplement: Supplementary file 1 — Supplementary Material 1 [file 10548_2023_975_MOESM1_ESM.docx]

**Electroencephalography-derived functional connectivity in sensorimotor networks in Post Stroke Fatigue**

Chi-Hsu Wu, William De Doncker, Annapoorna Kuppuswamy

Queen Square Institute of Neurology, University College London, WC1N 3BG

Abstract: 194 words

Full word count: 3607

Number of tables: 1

Number of figures: 1

**Corresponding author**

Annapoorna Kuppuswamy

Box 146, 33 Queen Square

Institute of Neurology,

London, WC1N 3BG

Email: [a.kuppuswamy@ucl.ac.uk](mailto:a.kuppuswamy@ucl.ac.uk)

Running title: Functional connectivity in post-stroke fatigue

Key words: Fatigue, Stroke, electroencephalography, small word index, beta band, connectivity, sensory network.

**Abstract**

Background: Poor suppression of anticipated sensory information from muscle contractions is thought to underlie high fatigue. Such diminished task-related sensory attenuation is reflected in resting state connectivity. Here we test the hypothesis ‘altered electroencephalography (EEG)-derived functional connectivity in somatosensory network in the beta band, is a signature of fatigue in post-stroke fatigue’.

Methods: In non-depressed, minimally impaired stroke survivors (n=29), with median disease duration of 5 years, resting state neuronal activity was measured using 64-channel EEG. Graph theory-based network analysis measure of functional connectivity via small-world index (SW) was calculated focusing on right and left motor (Brodmann areas 4, 6, 8, 9, 24 and 32) and sensory (Brodmann areas 1, 2, 3, 5, 7, 40 and 43) networks, in the beta (13-30 Hz) frequency range. Fatigue was measured using Fatigue Severity Scale - FSS (Stroke), with scores of >4, defined as high fatigue.

Results confirmed the working hypothesis, with high fatigue stroke survivors showing higher small-worldness in the somatosensory networks when compared to low fatigue.

Conclusion: High levels of small-worldness in somatosensory networks indicates altered processing of somesthetic input. Such altered processing would explain high effort perception within the sensory attenuation model of fatigue.

**Introduction**

Stroke, a result of vascular insufficiency to neurons present with fatigue as a significant symptom. The severity of stroke does not explain reported levels of fatigue (Kutlubaev, Duncan, and Mead 2012; van der Werf et al. 1998). Previously, we have proposed a sensory attenuation hypothesis of fatigue, based on poor suppression of anticipated sensory information –(Annapoorna Kuppuswamy 2017; 2022). Poor suppression of muscle sensory afferents results in assigning high effort to simple tasks which explains a significant proportion of post-stroke fatigue (Doncker et al. 2020) In visual and auditory perception, poor distractor suppression explains post-stroke fatigue (Annapoorna Kuppuswamy et al. 2022; Doncker and Kuppuswamy 2022). While other brain regions/networks have been implicated in fatigue such as parietal, pre-frontal and sub-cortical networks (Jaeger et al. 2019; Finke et al. 2015; Cotter et al. 2021), here we specifically focus on somatosensory and motor networks which have been implicated in PSF; to test the predictions of the sensory attenuation hypothesis that a dysfunction within the somatosensory networks underlies fatigue.

From a neuronal network functioning point of view, behaviour mirrors structural and functional changes in networks, which persist in resting state (Graziadio et al. 2010). Neuronal networks at rest express features that keep trace of their ability to perform the required behaviour (Kim and Kang 2018; Wahlheim et al. 2022; Doucet, Lam, and Griffin 2012; Y. Li et al. 2022; Liu et al. 2022). These features of networks at rest display alterations that reflect chronic symptoms (Porcaro et al. 2019). Both in post-stroke fatigue (PSF) and other disease where fatigue is significant, neurophysiological (A Kuppuswamy et al. 2015; Ondobaka et al. 2021; De Doncker, Brown, and Kuppuswamy 2021; Liepert et al. 2005; Morgante et al. 2011; Russo et al. 2017) and behavioural (A. Kuppuswamy et al. 2015; Annapoorna Kuppuswamy et al. 2016; De Doncker et al. 2020) findings support an altered resting state, specifically sensory network activity.

Ensembles of neurons that fire at specific frequencies and communicate with each other by synchronising their firing, comprise a neuronal network. To understand a network’s activity, the strength of synchronicity between various nodes is mapped using functional connectivity methods (Bullmore and Sporns 2009). Functional connectivity is defined as the temporal correlation or dependency between distinct neuronal groups and areas (Fingelkurts, Fingelkurts, and Kähkönen 2005; Rubinov and Sporns 2010). Such temporal correlation occurs in various frequency bands, with low frequencies associated with arousal, mid-range frequencies related to sensorimotor activity, and high frequencies representing higher order functions such as error detection and learning. With PSF proposed to be a problem of sensorimotor control, specifically arising from processing of incoming muscle related sensory information, we anticipated a fatigue related modulation of beta band frequency.

Here we investigate if a dysfunction of somatosensory networks underlies PSF, as demonstrated by changes in beta-band neuronal activity in sensory and motor networks at rest.

**Methods**

**Participants**

This study was approved by the London Bromley Research Ethics Committee (REC reference number: 16/LO/0714). Stroke survivors were recruited and tested at the Institute of Neurology, London, UK.

All stroke survivors were screened prior to the study based on the following criteria: first-time ischaemic or haemorrhagic stroke; stroke occurred at least 3 months prior to the study; no clinical diagnosis of any other neurological disorder; physically well recovered following their stroke defined as grip strength and manual dexterity of the affected hand being at least 60% of the unaffected hand assessed using a hand-held dynamometer and the nine-hole peg test (NHPT) respectively; not taking anti-depressants or any other medication that has a direct effect on the central nervous system; not clinically depressed with depression scores ≤ 11 assessed using the Hospital Anxiety and Depression Scale (HADS)(Snaith 2003).

Twenty-nine stroke survivors took part in the study (Table 1) and provided written informed consent in accordance with the Declaration of Helsinki. A formal sample-size calculation was not performed due to lack of pilot data. However, in previous studies, differences could be observed in the measure of resting state functional connectivity in as few as 10 subjects per group (Nordin et al. 2016).

**Fatigue**

Trait fatigue was quantified using Fatigue Severity Scale, FSS-7. An average score of one indicates no fatigue while an average score of seven indicates maximum fatigue (Krupp et al. 1989). High fatigue was defined as FSS-7>4 (Valko et al. 2008).

Control group: Healthy humans were not recruited for this study as a control group, specifically as the state of the brain is likely to have changed after an injury or establishment of a disease (Annapoorna Kuppuswamy 2023), therefore an ideal control group will be a within-disease control group. The stroke low (no) fatigue group was used as a control for this study.

**EEG recording**

Whole-scalp electroencephalography (EEG) data was recorded using 64-channel systems, ActiCap, Herrsching, Germany, and a BrainAmp, at rest, with eyes open and focusing on a fixation cross. Duration of recording was seven minutes. The 64 electrodes were positioned on the cap in accordance with the 10-20 international EEG electrode array. During online recordings, channels FCz and AFz were used as reference and ground respectively. Impedances were kept below 10 kΩ throughout the recording. The EEG signal was sampled at 1 kHz and visualized online using the BrainVision Recorder Software (BrainVision Recorder, Version 1.21.0102 Brain Products GmbH, Gilching, Germany).

**EEG Analysis**

EEG analyses were performed with a combination of EEGLAB(Delorme and Makeig 2004) and custom Matlab scripts. EEG data was down-sampled to 250 Hz and then band-pass filtered from 0.1 to 47 Hz using a finite impulse response filter. Noisy channels were identified and removed using automated procedures. EEG data was subsequently segmented into two second epochs, and epochs containing noisy data were identified as follows: the mean activity of all EEG channels was computed, and the threshold was set at ± 2 times the standard deviation of the mean activity. Epochs containing activity exceeding the threshold value were marked and subsequently removed. This left a total of 160 (± 15) two second epochs. To identify and remove ocular movements and blink artifacts from the EEG data, an independent component analysis (ICA) implemented within EEGLAB was used. ICA is a blind source decomposition algorithm that enables the separation of statistically independent sources from multichannel data (Jung et al. 2000). The components were subsequently visually inspected and those containing ocular movements or blink artifacts were removed. The previously removed channels were then interpolated back into the dataset and finally, the EEG data was re-referenced against the grand average of all scalp electrodes.

**Graph Theory Estimates**

*Functional Connectivity Analysis.* EEG connectivity analysis was carried out using the exact low-resolution electromagnetic tomography (eLORETA) software (The KEY Institute of Brain-Mind Research University Hospital of Psychiatry, Zurich; <http://www.uzh.ch/keyinst/NewLORETA/LORETA01.htm>). The eLORETA algorithm is a well-established linear inverse solution for EEG signals (R. D. Pascual-Marqui 2002).

Following whole brain sources reconstruction, connectivity was computed using the eLORETA software on four brain regions, divided into motor and sensory networks of the left and right hemisphere based on Broadmann areas (BAs). Each BA is a region of interest (ROI). The BAs that formed the motor network for both the left and right hemisphere included BA4, 6, 8, 9, 24 and 32, while the BAs that formed the sensory network for both the left and right hemisphere included BA1, 2, 3, 5, 7, 40 and 43.

Current density time series of all BAs within each of the four networks was computed in eLORETA and used to estimate the functional connectivity using the Lagged Linear Coherence (LagR) algorithm, not affected by volume conductance and low spatial resolution in each of the four networks (Roberto D. Pascual-Marqui 2007). Lagged Linear Coherence was computed for beta (13-30 Hz) band frequency.

*Graph Analysis.* A network is a mathematical representation of a real-world complex system and is defined by a collection of nodes (vertices) and links (edges) between pairs of nodes. Nodes in large-scale brain networks represent brain regions, while links represent anatomical or functional connections. Nodes should ideally represent brain regions with coherent patterns of anatomical or functional connections. The connectivity parameters extracted between all pairs of ROIs for each frequency band is in the form of a square matrix W, with dimensions equal to the number of ROIs. Each row and column within matrix W represent nodes, while the values within the matrix represent the strength of connection between each pair of nodes.

Once the networks of interest were constructed, the core measures of graph theory that summarize the aspects of segregation and integration of a network were computed using the Brain Connectivity Toolbox (Rubinov and Sporns 2010). Segregation refers to the degree to which network elements form individual and separate clusters and is measured by the clustering coefficient (*C*). Integration refers to the capacity of the network to become interconnected and exchange information and is measured by the parameter characteristic path length (*L*). The clustering coefficient and characteristic path length represent the efficiency of the network with respect to local and global connectedness respectively. Weighted clustering (Cw) coefficient and weighted characteristic path length (Lw) were computed as a measure of segregation and integration of the network as follows:

$Cw= \frac{C_{brain}}{C_{random}}$ and $Lw= \frac{L_{brain}}{L_{random}}$

Where C_brain_ and L_brain_ are the clustering coefficient and characteristic path length derived from the connectivity matrix of each participant. C_random_ and L_random_ are the mean values of the clustering coefficient and characteristic path length of 100 surrogate random networks that have the same basic characteristics as the original network that were derived by randomly rewiring the nodes using the original network edge weights. The random networks preserve the same basic characteristics as the original network such as the number of nodes and edge weights. The random networks were constructed by Brain Connectivity Toolbox^29^. A measure of network small-worldness (Sw) was therefore defined as the ratio between Cw and Lw; the ratio between local connectedness and the global integration of the network.

$$Sw= \frac{Cw}{Lw}$$

When Sw has a value of approximately 1, a network is said to have “small-world properties” meaning a good combination of high levels of local clustering among nodes and proper paths that globally link all network nodes (all nodes of a large system are linked through relatively few intermediate steps). Sw values greater than 1 suggest high levels of local clustering among nodes and many short paths that globally link all nodes of the network, while Sw values less than 1 suggest poor local connectivity and stunted connections.

**Statistical Analysis**

All statistical analysis was performed using R (RStudio Version 1.2.5033). Spearman rank correlations identified associations between trait fatigue (FSS-7) and demographic variables (age, grip strength, NHPT, HADS – Depression, HADS – Anxiety and Time Post-Stroke). Wilcoxon rank sum tests identified association between trait fatigue (FSS-7 scores) and categorical measures (sex, hemisphere affected and type of stroke). The effect of vascular territory on FSS was not analysed as there were too few in each group. Shapiro-Wilk’s test of normality assessed distribution of dependent variable and Levene’s test assessed homogeneity of variances.

In normally distributed variables, a three-way mixed ANOVA was performed to evaluate the effects of fatigue (between subject factor: Stroke_Low_, Stroke_High_) and sex (between subject factor: Male, Female) on small worldness within the beta frequency band (dependent variable), for two networks (sensory and motor) and two hemispheres (left and right). Greenhouse-Geisser epsilon adjustment corrected any deviations from sphericity. Post-hoc pairwise comparisons (t-tests) with Bonferroni adjustment was performed to identify main effects.

**Results**

**Participant Demographics**

Twenty-nine stroke survivors completed the study (11 females and 18 males). The median FSS-7 score was 5.29 (IQR=2.57) in females and 2.50 (IQR=2.46) in males. The Wilcoxon test showed that the difference in FSS-7 score was marginally non-significant (p=0.05, effect size=0.37). Spearman rank correlations between trait fatigue (FSS-7) and all continuous demographic measures revealed a significant positive association between trait fatigue and HADS-Depression (Spearman ρ =0.41, p=0.03), while no other variable correlated with trait fatigue (Age: Spearman ρ = 0.02, p = 0.91; Grip strength: Spearman ρ = -0.23, p = 0.24; NHPT: Spearman ρ = -0.25, p = 0.19; HADS-Anxiety: Spearman ρ = 0.35, p = 0.06).

|  | Fatigue Group | |
| --- | --- | --- |
| Variable | Low Fatigue, N = 16^1^ | High Fatigue, N = 13^1^ |
| **FSS-7** | 2.1 (1.4,2.4) | 5.6 (5.3,6.0) |
| **Age (years)** | 61.7 (55.6,64.8) | 62.9 (56.1,68.1) |
| **Sex** |  |  |
| Male | 13 | 5 |
| Female | 3 | 8 |
| **Grip Strength (% unaffected hand)** | 98.4 (89.9,107.3) | 92.3 (79.7,103.3) |
| **NHPT (% unaffected hand)** | 94.3 (86.0,105.) | 87.7 (69.4,94.8) |
| **SDMT** | 1.0 (0.8,1.2) | 0.8 (0.5,1.0) |
| **HADS - Anxiety** | 4.0 (2.8,7.3) | 9.0 (3.0,10.0) |
| **HADS - Depression** | 4.0 (3.0,5.0) | 7.0 (3.0,9.0) |
| **Hemisphere Affected** |  |  |
| Left | 10 | 6 |
| Right | 6 | 7 |
| **Type of Stroke** |  |  |
| Ischaemic | 14 | 12 |
| Hemorrhagic | 2 | 1 |
| **Vascular Territory Affected** |  |  |
| MCA | 8 | 8 |
| PCA | 1 | 1 |
| Brainstem/Cerebellum | 3 | 3 |
| **Time Post-Stroke (years)** | 5.3 (4.2,6.8) | 7.4 (5.4,11.1) |
| ^1^Median (25%,75%); n | | |

**Table 1**: This table provides the demographic and lesion information of the stroke survivors cohort.

**Clinical Characteristics**

There were no confirmed MRI lesions in any of the stroke survivors in the study. The association between trait fatigue (FSS-7) and the clinical characteristics of the stroke was assessed across all stroke survivors. The median FSS-7 score in those with right hemisphere strokes was 4.43 (IQR=3.00) and 2.71 (IQR=3.75) in those with left hemisphere strokes (Wilcoxon test: p=0.50, effect size r=0.13). The median FSS-7 score in those with ischemic strokes was 2.93 (IQR=3.46) and 3.86 (IQR=2.29) in those with hemorrhagic strokes (Wilcoxon test: p=0.51, effect size r=0.13). Regarding the vascular territory affected, the data from five stroke survivors was missing as the clinical notes could not be retrieved. The median FSS-7 score in those where the MCA was affected was 3.36 (IQR=3.25), the median FSS-7 score in those where the PCA was affected was 3.07 (IQR=2.07), while the median FSS-7 score in those where the Brainstem/Cerebellum was affected was 4.21 (IQR=2.68) (Kruskal-Wallis test: p=0.57, effect size η^2^=-0.04). A spearman rank correlation between FSS-7 and the Time Post-Stroke at which the participants took part in the study showed no significant association (spearman ρ=0.08, p=0.67). Any meaningful interpretation of the effect of the type of stroke and vascular territory affected on FSS-7 in the current cohort of stroke survivors is difficult given the skewed numbers.

**Small Worldness**

Across the two networks (sensory/motor) and hemispheres (left/right) there were two extreme outliers (both in the motor network of the right hemisphere). After exclusion of outliers, the data was normally distributed, (p > 0.05) and variances were homogenous (p > 0.05). The three-way-ANOVA revealed a main effect of network type (F_(1,23)_=15.59, p<0.01, η^2^=0.13) and a significant interaction between network type and fatigue level (F_(1,23)_=16.79, p<0.01, η^2^=0.14) on small worldness in beta frequency band. There was also a significant three way interaction between network type, fatigue level and sex (F_(1,23)_=5.09, p=0.03, η^2^=0.05), however there was no significant two way interaction between sex and network type (F_(1,23)_=0.65, p=0.43, η^2^=0.006) nor between sex and fatigue (F_(1,23)_=1.05, p=0.32, η^2^=0.009). Post-hoc multiple pairwise comparisons revealed a significant difference in small worldness within the beta frequency band (Figure 1) between the Stroke_Low_ and Stroke_High_ groups in the sensory network of the right hemisphere (p=0.01), driven by the difference in female participants (P=0.0127). There was also a significant difference between Stroke_Low_ and Stroke_High_ in left and right motor networks (p=0.02 and p=0.03 respectively), with females driving the difference in the right hemisphere (P=0.0138).


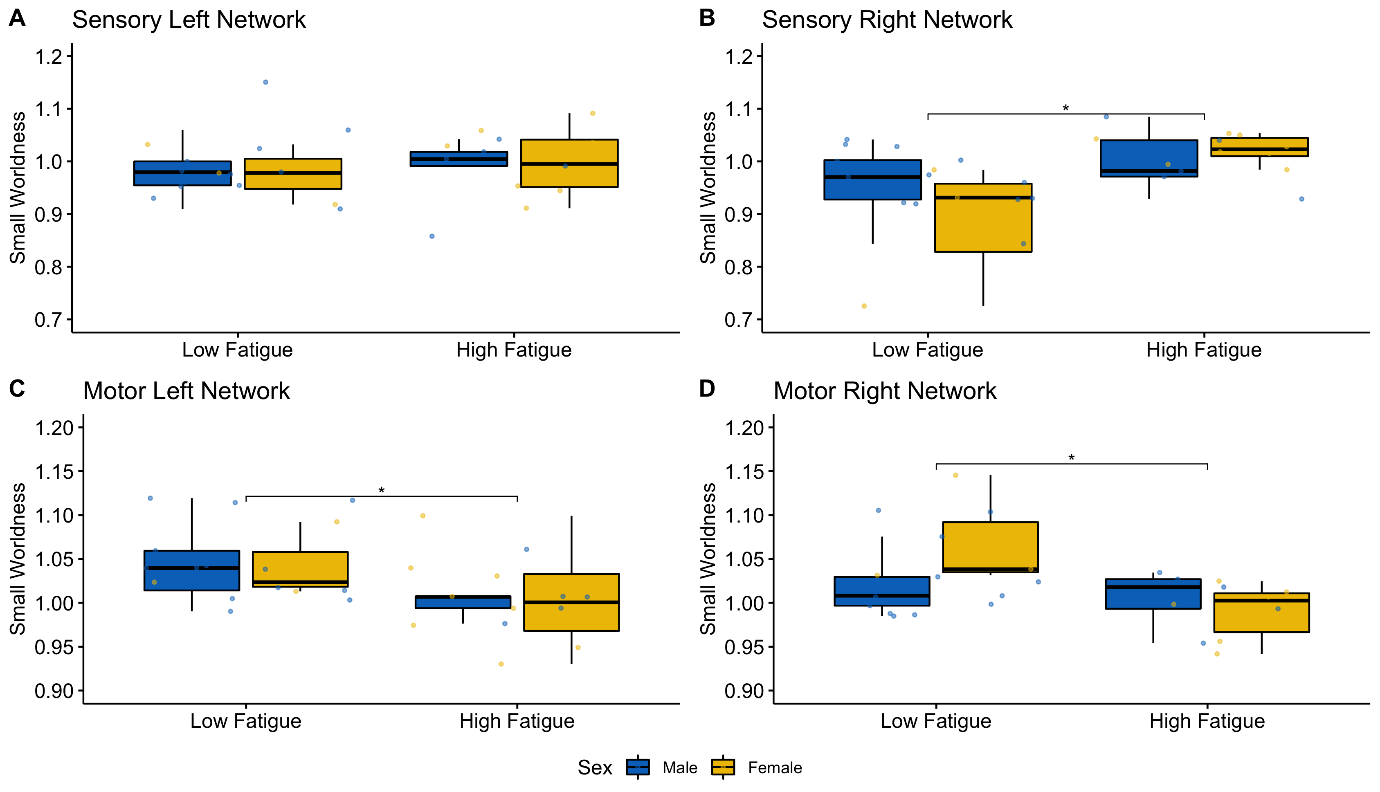


**Figure 1**. Functional Connectivity of the Sensory and Motor Networks in the beta band frequency. The value of Small Worldness across the two fatigue groups both in males and females (Stroke_Male_ in blue and Stroke_Female_ in yellow) is displayed using boxplots for the sensory network in the left (A) and right (B) hemispheres and for motor networks in the left (C) and right (D) hemispheres. Significant differences between fatigue groups are indicated using asterisks (* = p < 0.05). In the right motor and sensory networks, the difference is driven by females.

**Discussion**

In twenty-nine patients with stroke, we show that those with high levels of trait fatigue exhibited significantly higher levels of small worldness in the sensory networks and significantly lower levels of small worldness in the motor networks in the beta band frequency (13-30Hz). There was no association between clinical features of stroke, and trait fatigue which confirms previous findings (Kutlubaev, Duncan, and Mead 2012). As investigated by small worldness, functional brain connectivity simultaneously reconciles the opposing demands of functional integration and segregation. The small-world index reflects the balance of functionally specialized (segregated) modules with a robust number of intermodular (integrating) links. Here we observed that fatigue is paralleled by the alteration of small-world organization in sensory and motor networks.

The observed changes in sensory networks are lateralised. A possible trivial explanation could be that the small numbers have resulted in one side reaching significance and not the other. If the observed lateralisation is not due to limited numbers, ‘hyper-connectivity’ in right sensory networks is in keeping with recent findings of a shift towards right hemispheric dominance in sensorimotor networks in those with high post-stroke fatigue (Ondobaka et al. 2021). Healthy brains exhibit left-hemispheric dominance (Netz, Ziemann, and Hömberg 1995; Giovannelli et al. 2009) and a shift towards right dominance has been observed in several psychiatric diseases including depression (Lefaucheur et al. 2008). A third possible non-trivial explanation could be the small difference in the number of left hemispheric strokes in the low fatigue group, however, this is unlikely as previous studies show shift in hemispheric dominance regardless of the side of stroke (Ondobaka et al. 2021).

Beta band activity is commonly known as the sensorimotor ‘idling’ rhythm seen in all cortical and sub-cortical motor areas at rest. Movement desynchronises beta band oscillations which lead to the idea that beta frequency is the rhythm of rest for sensorimotor areas. Recent proposals suggest that beta band activity may not simply reflect a lack of movement but is rather an indicator for maintenance of sensorimotor status quo (Engel and Fries 2010). During periods of spontaneous enhancement in resting beta band activity, movements are slower, than when resting beta activity is lower (Gilbertson et al. 2005). In light of such findings, the current results indicate that those with high fatigue are likely to have slowed movements. In post-stroke fatigue, while there is no difference in reaction times, there is slowing of movements (A. Kuppuswamy et al. 2015), perhaps because there is a resistance to change sensorimotor status quo as reflected by enhanced beta rhythm small-worldness shown in this study.

It is well-established that fatigue is only marginally associated with motor and cognitive deficits (Ingles, Eskes, and Phillips 1999; Winward et al. 2009). However, despite good functional ability, markers of poor behavioural flexibility is associated with high fatigue (De Doncker, Brown, and Kuppuswamy 2021; Morgante et al. 2011). Enhanced sensory, and diminished motor network functional connectivity that seeks to maintain a sensory state, thereby making new sensory states less desirable, lends further support to the idea of poor behavioural flexibility underpinning high fatigue.

In this study we provide evidence for alteration in somatosensory processing, which may indicate a possible mechanism that drives fatigue is poor somatosensory processing. Both in post-stroke fatigue and other neurological conditions such as MS, there are several reports of altered resting state connectivity (Ondobaka et al. 2021; Woodward et al. 2019; Bisecco et al. 2018; Jaeger et al. 2019; Stefancin et al. 2019; Cotter et al. 2021). In stroke, suggestions of parietal hypoconnectivity and frontal hyper connectivity (Cotter et al. 2021) with reversed inter-hemispheric balance of connectivity (Ondobaka et al. 2021), are implicated in manifestation of fatigue. In MS, changes in default mode network (Jaeger et al. 2019) and involvement of striatal circuits involved in movement, sensation and motivation (Bisecco et al. 2018) have all been implicated in development of fatigue. While several brain regions have been implicated in both diseases as the core regions involved in fatigue, very few of the studies performed hypothesis driven analysis on resting state activity. In the present study, we hypothesised that attention to somatosensory input in not suppressed as normal [indicated by high perceived effort during muscle contraction (Doncker et al. 2020)] which will be reflected in the resting state both in sensory and motor networks. Hyper-connectivity in sensory networks, commonly seen as a marker of tendency to maintain sensory states quo, and resultant hypoconnectivity in motor networks that indicate lower M1 excitability (Annapoorna Kuppuswamy et al. 2015) supports the premise that poor somatosensory attenuation underpins high PSF. In summary, increased small world-ness in somatosensory networks suggests a propensity to maintain the status quo i.e. rest; an increased propensity to inaction (rest) translates into an increase in the effort needed to initiate an action; increased effort results in fatigue.

The high fatigue group had a disproportionately high number of females when compared to the low fatigue group. Previous studies have reported greater incidence of fatigue in female stroke survivors (Cumming et al. 2016) and sex significantly influences measures of resting state connectivity (Stumme et al. 2020). While difference in incidence of fatigue was previously attributed to factors such as reporting biases influenced by sociocultural factors, the difference in resting state connectivity between males and females with high fatigue seen in this study opens up the possibility of a biological basis for differences in incidence of PSF. The influence of sex on resting state connectivity regardless of fatigue or stroke is unlikely to be the driver of the differences seen here, as sensorimotor networks are less likely to be influenced by sex (L. Li, Cui, and Wang 2022), unlike other brain networks. While further speculation on differences in biological mechanisms driving fatigue in males and females is beyond the scope of this paper, future mechanistic studies in fatigue must consider the possibility of sex being a confounding factor in interpretation of findings, and also directly study differences between males and females in PSF.

Limitations While the hypothesis-driven approach of this study is a strength, it could also be a limitation. No neural network in the brain operates in isolation and the influences of other networks on sensorimotor areas of the brain are also likely influenced by the differences seen in this study, which needs further investigation. The relatively small numbers of participants in this study is further highlighted by the differences seen between sexes in this study. Lesion location does not influence PSF incidence or severity, however, to definitively exclude influence of lesion location on neural network activity relevant to fatigue, greater numbers in each lesion type is warranted.

**Conclusion**

Chronic fatigue is a feature of several established long-term disorders. However, there is no principled framework to understand the mechanisms of fatigue. In this paper, we demonstrate sensorimotor network activity is altered in line with the predictions of the sensory attenuation model of fatigue in chronic stroke survivors. This is a promising framework which could explain altered connectivity seen in other neurological disorders with long-term fatigue and future work must focus on exploring sensory attenuation in chronic fatigue.

**Funding**

Wellcome trust: 202346/Z/16/Z

**Data availability**

The processed EEG data is available on request from [a.kuppuswamy@ucl.ac.uk](mailto:a.kuppuswamy@ucl.ac.uk)

**Acknowledgements**

We would like to thank Prof Franca Tecchio, Dr Pierepaolo Croche and Dr Massimo Bertoli for the many discussions and input into this manuscript. While the intention of this paper was to include a multiple sclerosis resting state dataset from Prof Tecchio’s group, this did not come to fruition due to several incompatibilities. Nevertheless, the highly enthusiastic discussions were key to bringing this paper to print and must be acknowledged.

**References**

Bisecco, Alvino, Federica Di Nardo, Renato Docimo, Giuseppina Caiazzo, Alessandro d’Ambrosio, Simona Bonavita, Rocco Capuano, et al. 2018. “Fatigue in Multiple Sclerosis: The Contribution of Resting-State Functional Connectivity Reorganization.” *Multiple Sclerosis (Houndmills, Basingstoke, England)* 24 (13): 1696–1705. https://doi.org/10.1177/1352458517730932.

Bullmore, Ed, and Olaf Sporns. 2009. “Complex Brain Networks: Graph Theoretical Analysis of Structural and Functional Systems.” *Nature Reviews. Neuroscience* 10 (3): 186–98. https://doi.org/10.1038/nrn2575.

Cotter, Georgia, Mohamed Salah Khlif, Laura Bird, Mark E Howard, Amy Brodtmann, and Natalia Egorova-Brumley. 2021. “Post-Stroke Fatigue Is Associated with Resting State Posterior Hypoactivity and Prefrontal Hyperactivity.” *International Journal of Stroke: Official Journal of the International Stroke Society*, September, 17474930211048324. https://doi.org/10.1177/17474930211048323.

Cumming, Toby B., Marcie Packer, Sharon F. Kramer, and Coralie English. 2016. “The Prevalence of Fatigue after Stroke: A Systematic Review and Meta-Analysis.” *International Journal of Stroke: Official Journal of the International Stroke Society* 11 (9): 968–77. https://doi.org/10.1177/1747493016669861.

De Doncker, William, Katlyn E. Brown, and Annapoorna Kuppuswamy. 2021. “Influence of Post-Stroke Fatigue on Reaction Times and Corticospinal Excitability during Movement Preparation.” *Clinical Neurophysiology* 132 (1): 191–99. https://doi.org/10.1016/j.clinph.2020.11.012.

De Doncker, William, Lucie Charles, Sasha Ondobaka, and Annapoorna Kuppuswamy. 2020. “Exploring the Relationship between Effort Perception and Post-Stroke Fatigue.” *Neurology*, October. https://doi.org/10.1212/WNL.0000000000010985.

Delorme, Arnaud, and Scott Makeig. 2004. “EEGLAB: An Open Source Toolbox for Analysis of Single-Trial EEG Dynamics Including Independent Component Analysis.” *Journal of Neuroscience Methods* 134 (1): 9–21. https://doi.org/10.1016/j.jneumeth.2003.10.009.

Doncker, William De, Lucie Charles, Sasha Ondobaka, and Annapoorna Kuppuswamy. 2020. “Exploring the Relationship between Effort Perception and Poststroke Fatigue.” *Neurology* 95 (24): e3321–30. https://doi.org/10.1212/WNL.0000000000010985.

Doncker, William De, and Annapoorna Kuppuswamy. 2022. “The Cocktail Party Effect in Post-Stroke Fatigue: An EEG Study.” bioRxiv. https://doi.org/10.1101/2022.03.17.484808.

Doucet, Barbara M., Amy Lam, and Lisa Griffin. 2012. “Neuromuscular Electrical Stimulation for Skeletal Muscle Function.” *The Yale Journal of Biology and Medicine* 85 (2): 201–15.

Engel, Andreas K., and Pascal Fries. 2010. “Beta-Band Oscillations--Signalling the Status Quo?” *Current Opinion in Neurobiology* 20 (2): 156–65. https://doi.org/10.1016/j.conb.2010.02.015.

Fingelkurts, Andrew A., Alexander A. Fingelkurts, and Seppo Kähkönen. 2005. “Functional Connectivity in the Brain--Is It an Elusive Concept?” *Neuroscience and Biobehavioral Reviews* 28 (8): 827–36. https://doi.org/10.1016/j.neubiorev.2004.10.009.

Finke, C., J. Schlichting, S. Papazoglou, M. Scheel, A. Freing, C. Soemmer, L. M. Pech, et al. 2015. “Altered Basal Ganglia Functional Connectivity in Multiple Sclerosis Patients with Fatigue.” *Multiple Sclerosis (Houndmills, Basingstoke, England)* 21 (7): 925–34. https://doi.org/10.1177/1352458514555784.

Gilbertson, Thomas, Elodie Lalo, Louise Doyle, Vincenzo Di Lazzaro, Beatrice Cioni, and Peter Brown. 2005. “Existing Motor State Is Favored at the Expense of New Movement during 13-35 Hz Oscillatory Synchrony in the Human Corticospinal System.” *Journal of Neuroscience* 25 (34): 7771–79. https://doi.org/10.1523/JNEUROSCI.1762-05.2005.

Giovannelli, Fabio, Alessandra Borgheresi, Fabrizio Balestrieri, Gaetano Zaccara, Maria Pia Viggiano, Massimo Cincotta, and Ulf Ziemann. 2009. “Modulation of Interhemispheric Inhibition by Volitional Motor Activity: An Ipsilateral Silent Period Study.” *The Journal of Physiology* 587 (Pt 22): 5393–5410. https://doi.org/10.1113/jphysiol.2009.175885.

Graziadio, Sara, Anna Basu, Leo Tomasevic, Filippo Zappasodi, Franca Tecchio, and Janet A. Eyre. 2010. “Developmental Tuning and Decay in Senescence of Oscillations Linking the Corticospinal System.” *The Journal of Neuroscience: The Official Journal of the Society for Neuroscience* 30 (10): 3663–74. https://doi.org/10.1523/JNEUROSCI.5621-09.2010.

Ingles, J L, G A Eskes, and S J Phillips. 1999. “Fatigue after Stroke.” *Archives of Physical Medicine and Rehabilitation* 80 (2): 173–78.

Jaeger, Sven, Friedemann Paul, Michael Scheel, Alexander Brandt, Josephine Heine, Daniel Pach, Claudia M. Witt, Judith Bellmann-Strobl, and Carsten Finke. 2019. “Multiple Sclerosis-Related Fatigue: Altered Resting-State Functional Connectivity of the Ventral Striatum and Dorsolateral Prefrontal Cortex.” *Multiple Sclerosis (Houndmills, Basingstoke, England)* 25 (4): 554–64. https://doi.org/10.1177/1352458518758911.

Jung, Tzyy-Ping, Scott Makeig, Colin Humphries, Te-Won Lee, Martin J. McKEOWN, Vicente Iragui, and Terrence J. Sejnowski. 2000. “Removing Electroencephalographic Artifacts by Blind Source Separation.” *Psychophysiology* 37 (2): 163–78. https://doi.org/10.1111/1469-8986.3720163.

Kim, Jinhee, and Eunjoo Kang. 2018. “Strength of Resting-State Functional Connectivity Associated with Performance-Adjustment Ability.” *Behavioural Brain Research* 347 (July): 377–84. https://doi.org/10.1016/j.bbr.2018.02.024.

Krupp, Lauren B., Nicholas G. LaRocca, Joanne Muir-Nash, and Alfred D. Steinberg. 1989. “The Fatigue Severity Scale: Application to Patients With Multiple Sclerosis and Systemic Lupus Erythematosus.” *Archives of Neurology* 46 (10): 1121–23. https://doi.org/10.1001/archneur.1989.00520460115022.

Kuppuswamy, A., E. V. Clark, K. S. Sandhu, J. C. Rothwell, and N. S. Ward. 2015. “Post-Stroke Fatigue: A Problem of Altered Corticomotor Control?” *Journal of Neurology, Neurosurgery, and Psychiatry* 86 (8): 902–4. https://doi.org/10.1136/jnnp-2015-310431.

Kuppuswamy, A, Ella V. Clark, Isobel F. Turner, John C. Rothwell, and Nick S. Ward. 2015. “Post-Stroke Fatigue: A Deficit in Corticomotor Excitability?” *Brain: A Journal of Neurology* 138 (Pt 1): 136–48. https://doi.org/10.1093/brain/awu306.

Kuppuswamy, Annapoorna. 2017. “The Fatigue Conundrum.” *Brain* 140 (8): 2240–45. https://doi.org/10.1093/brain/awx153.

Kuppuswamy, Annapoorna. 2022. “The Neurobiology of Pathological Fatigue: New Models, New Questions.” *The Neuroscientist: A Review Journal Bringing Neurobiology, Neurology and Psychiatry* 28 (3): 238–53. https://doi.org/10.1177/1073858420985447.

Kuppuswamy, Annapoorna. 2023. “On the Importance of Not Comparing Apples with Pears.” *European Journal of Neurology* 30 (2): 557. https://doi.org/10.1111/ene.15587.

Kuppuswamy, Annapoorna, Ella Clark, John Rothwell, and Nick S. Ward. 2016. “Limb Heaviness: A Perceptual Phenomenon Associated With Poststroke Fatigue?” *Neurorehabilitation and Neural Repair* 30 (4): 360–62. https://doi.org/10.1177/1545968315597071.

Kuppuswamy, Annapoorna, Ella V. Clark, Isobel F. Turner, John C. Rothwell, and Nick S. Ward. 2015. “Post-Stroke Fatigue: A Deficit in Corticomotor Excitability?” *Brain: A Journal of Neurology* 138 (Pt 1): 136–48. https://doi.org/10.1093/brain/awu306.

Kuppuswamy, Annapoorna, Anthony M. Harris, William De Doncker, Adrian Alexander, and Nilli Lavie. 2022. “Diminished Distractor Filtering with Increased Perceptual Load and Sustained Effort Explains Attention Deficit in Post-Stroke Fatigue.” bioRxiv. https://doi.org/10.1101/2022.03.17.484709.

Kutlubaev, M. A., F. H. Duncan, and G. E. Mead. 2012. “Biological Correlates of Post-Stroke Fatigue: A Systematic Review.” *Acta Neurologica Scandinavica* 125 (4): 219–27. https://doi.org/10.1111/j.1600-0404.2011.01618.x.

Lefaucheur, J. P., B. Lucas, F. Andraud, J. Y. Hogrel, F. Bellivier, A. Del Cul, A. Rousseva, M. Leboyer, and M. L. Paillère-Martinot. 2008. “Inter-Hemispheric Asymmetry of Motor Corticospinal Excitability in Major Depression Studied by Transcranial Magnetic Stimulation.” *Journal of Psychiatric Research* 42 (5): 389–98. https://doi.org/10.1016/j.jpsychires.2007.03.001.

Li, Leinian, Zhijun Cui, and Li Wang. 2022. “A More Female-Characterized Resting-State Brain: Graph Similarity Analyses of Sex Influence on the Human Brain Intrinsic Functional Network.” *Brain Topography* 35 (3): 341–51. https://doi.org/10.1007/s10548-022-00900-5.

Li, Yanyan, Lihao Yang, Lihua Li, Yuanjun Xie, and Peng Fang. 2022. “The Resting-State Cerebro-Cerebellar Function Connectivity and Associations with Verbal Working Memory Performance.” *Behavioural Brain Research* 417 (January): 113586. https://doi.org/10.1016/j.bbr.2021.113586.

Liepert, J, D Mingers, C Heesen, T Bäumer, and C Weiller. 2005. “Motor Cortex Excitability and Fatigue in Multiple Sclerosis: A Transcranial Magnetic Stimulation Study.” *Multiple Sclerosis (Houndmills, Basingstoke, England)* 11 (3): 316–21.

Liu, Yadong, Xi Ren, Mei Zeng, Jiwen Li, Xiaolin Zhao, Xuehan Zhang, and Juan Yang. 2022. “Resting-State Dynamic Functional Connectivity Predicts the Psychosocial Stress Response.” *Behavioural Brain Research* 417 (January): 113618. https://doi.org/10.1016/j.bbr.2021.113618.

Morgante, Francesca, Vincenzo Dattola, Domenica Crupi, Margherita Russo, Vincenzo Rizzo, Maria Felice Ghilardi, Carmen Terranova, Paolo Girlanda, and Angelo Quartarone. 2011. “Is Central Fatigue in Multiple Sclerosis a Disorder of Movement Preparation?” *Journal of Neurology* 258 (2): 263–72. https://doi.org/10.1007/s00415-010-5742-x.

Netz, J., U. Ziemann, and V. Hömberg. 1995. “Hemispheric Asymmetry of Transcallosal Inhibition in Man.” *Experimental Brain Research* 104 (3): 527–33. https://doi.org/10.1007/BF00231987.

Nordin, Love Engström, Marika Christina Möller, Per Julin, Aniko Bartfai, Farouk Hashim, and Tie-Qiang Li. 2016. “Post MTBI Fatigue Is Associated with Abnormal Brain Functional Connectivity.” *Scientific Reports* 6 (February): 21183. https://doi.org/10.1038/srep21183.

Ondobaka, Sasha, William De Doncker, Nick Ward, and Annapoorna Kuppuswamy. 2021. “Neural Effective Connectivity Explains Subjective Fatigue in Stroke.” *Brain: A Journal of Neurology*, November, awab287. https://doi.org/10.1093/brain/awab287.

Pascual-Marqui, R. D. 2002. “Standardized Low-Resolution Brain Electromagnetic Tomography (SLORETA): Technical Details.” *Methods and Findings in Experimental and Clinical Pharmacology* 24 Suppl D: 5–12.

Pascual-Marqui, Roberto D. 2007. “Instantaneous and Lagged Measurements of Linear and Nonlinear Dependence between Groups of Multivariate Time Series: Frequency Decomposition.” *ArXiv:0711.1455 [Stat]*, November. http://arxiv.org/abs/0711.1455.

Porcaro, Camillo, Carlo Cottone, Andrea Cancelli, Paolo M. Rossini, Giancarlo Zito, and Franca Tecchio. 2019. “Cortical Neurodynamics Changes Mediate the Efficacy of a Personalized Neuromodulation against Multiple Sclerosis Fatigue.” *Scientific Reports* 9 (1): 18213. https://doi.org/10.1038/s41598-019-54595-z.

Rubinov, Mikail, and Olaf Sporns. 2010. “Complex Network Measures of Brain Connectivity: Uses and Interpretations.” *NeuroImage* 52 (3): 1059–69. https://doi.org/10.1016/j.neuroimage.2009.10.003.

Russo, M., A. Calamuneri, A. Cacciola, L. Bonanno, A. Naro, V. Dattola, E. Sessa, et al. 2017. “Neural Correlates of Fatigue in Multiple Sclerosis: A Combined Neurophysiological and Neuroimaging Approach (R1).” *Archives Italiennes De Biologie* 155 (3): 142–51.

Snaith, R Philip. 2003. “The Hospital Anxiety And Depression Scale.” *Health and Quality of Life Outcomes* 1 (August): 29. https://doi.org/10.1186/1477-7525-1-29.

Stefancin, Patricia, Sindhuja T. Govindarajan, Lauren Krupp, Leigh Charvet, and Timothy Q. Duong. 2019. “Resting-State Functional Connectivity Networks Associated with Fatigue in Multiple Sclerosis with Early Age Onset.” *Multiple Sclerosis and Related Disorders* 31 (June): 101–5. https://doi.org/10.1016/j.msard.2019.03.020.

Stumme, Johanna, Christiane Jockwitz, Felix Hoffstaedter, Katrin Amunts, and Svenja Caspers. 2020. “Functional Network Reorganization in Older Adults: Graph-Theoretical Analyses of Age, Cognition and Sex.” *NeuroImage* 214 (July): 116756. https://doi.org/10.1016/j.neuroimage.2020.116756.

Valko, Philipp O., Claudio L. Bassetti, Konrad E. Bloch, Ulrike Held, and Christian R. Baumann. 2008. “Validation of the Fatigue Severity Scale in a Swiss Cohort.” *Sleep* 31 (11): 1601–7.

Wahlheim, Christopher N., Alexander P. Christensen, Zachariah M. Reagh, and Brittany S. Cassidy. 2022. “Intrinsic Functional Connectivity in the Default Mode Network Predicts Mnemonic Discrimination: A Connectome-Based Modeling Approach.” *Hippocampus* 32 (1): 21–37. https://doi.org/10.1002/hipo.23393.

Werf, S. P. van der, P. J. Jongen, G. J. Lycklama à Nijeholt, F. Barkhof, O. R. Hommes, and G. Bleijenberg. 1998. “Fatigue in Multiple Sclerosis: Interrelations between Fatigue Complaints, Cerebral MRI Abnormalities and Neurological Disability.” *Journal of the Neurological Sciences* 160 (2): 164–70.

Winward, Charlotte, Catherine Sackley, Ziyah Metha, and Peter M Rothwell. 2009. “A Population-Based Study of the Prevalence of Fatigue after Transient Ischemic Attack and Minor Stroke.” *Stroke; a Journal of Cerebral Circulation* 40 (3): 757–61. https://doi.org/10.1161/STROKEAHA.108.527101.

Woodward, K. E., H. L. Carlson, A. Kuczynski, J. Saunders, J. Hodge, and A. Kirton. 2019. “Sensory-Motor Network Functional Connectivity in Children with Unilateral Cerebral Palsy Secondary to Perinatal Stroke.” *NeuroImage. Clinical* 21: 101670. https://doi.org/10.1016/j.nicl.2019.101670.
